# Depressive and anxiety symptoms among higher vocational nursing interns in Ningbo, China: a cross-sectional study

**DOI:** 10.3389/fpsyg.2025.1450071

**Published:** 2026-01-22

**Authors:** Yuchen Ying, Xiaomei Chen, Li Wang

**Affiliations:** 1 Ningbo College of Health Sciences, Ningbo, Zhejiang, China; 2 Department of Psychosomatic Medicine, The First Affiliated Hospital of Ningbo University, Ningbo, Zhejiang, China

**Keywords:** higher vocational nursing students, nursing intern, depressive symptoms, anxiety symptoms, cross-sectional study

## Abstract

**Background:**

Extensive studies have revealed that undergraduate nursing students experience several mental health problems during internships. Nevertheless, the mental health status of higher vocational nursing interns, especially 2-year college interns, has been neglected. This study examined the prevalence of depressive and anxiety symptoms, along with associated risk factors, among higher vocational nursing interns, and, for the first time, compared symptom prevalence between junior college and senior college interns, as well as between 2-year and 3-year college interns.

**Methods:**

This cross-sectional study enrolled nursing interns from a higher vocational college in Ningbo, China. Mental health status was assessed using the Chinese versions of the Self-Rating Depression Scale (SDS) and Self-Rating Anxiety Scale (SAS). Multivariable logistic regression analyses were performed to identify the main factors associated with depressive and anxiety symptoms by calculating multivariable-adjusted odds ratios (ORs) and 95% confidence intervals (CI).

**Results:**

The overall prevalence of depressive and anxiety symptoms were 36.93% (95% CI: 32.72–41.24%) and 19.56% (95% CI: 16.26–23.31%), respectively. The prevalence of depressive symptoms (*P* = 0.012) and anxiety symptoms (*P* < 0.001) was significantly higher among 2-year college students than among 3-year college students. Factors such as younger age (OR: 1.26), residence in rural areas (OR: 1.80), low family function (OR: 2.96), internships in third-class hospitals (OR: 1.67), and enrollment as a 2-year college student (OR: 1.62) significantly increased the risk of depressive symptoms. Rural areas (OR: 3.337), low family functioning (OR: 2.905), internships in third-class hospitals (OR: 1.420), and being a 2-year college student (OR: 4.231) significantly increased the risk of anxiety symptoms during the internship.

**Conclusion:**

The results of this study suggest that psychological problems have been dramatic among Chinese higher vocational nursing students, especially the 2-year college interns, during internships. Our findings provide information to aid in promoting the mental health of this vulnerable population.

## 1 Introduction

Since the 1980s, China has established a comprehensive nursing education system, which is composed of 3-year secondary diploma programs, 3-year advanced diploma programs, and 4-year or 5-year baccalaureate programs (Chen et al., 2020). Higher vocational nursing students in China can be divided into two categories: first, students aged 18–19 years who have completed the 12-year general education and then complete their 3-year advanced diploma programs in a higher vocational college (i.e., 3-year college nursing students). Second, students aged 15–16 years who complete 9 years of general education may enter a 2-year secondary diploma program in a secondary vocational school, followed by a 2-year advanced diploma program in a higher vocational college (i.e., 2-year college nursing students) (You et al., 2015). These students complete a 5-year integrated curriculum combining secondary and higher vocational courses, but with a compressed total number of class hours. As a result, they possess a relatively weak foundation and must balance basic education with vocational training within a limited time. In contrast, 3-year college students typically complete 3 years of general education before beginning a 3-year professional nursing program. This curriculum is more systematic, providing students with a stronger cultural and academic foundation upon entry. However, while higher vocational nursing students account for more than 60% of the total number of nursing students in China (Rodgers et al., 2015), to date, extensive studies only revealed that undergraduate nursing students in China experience various mental health problems (Wang et al., 2023; Niu et al., 2023). Only a few studies have focused on the mental health of nursing students in higher vocational colleges, especially the comparison of these two different categories of higher vocational nursing students.

At a higher vocational college in China, nursing students spend their first 2 years in college taking theoretical courses, and their third year in a hospital internship (Chen et al., 2020). Growing evidence suggests that hospital internships are fundamental to nursing education and contribute to acquiring and sustainable developing nursing skills (Chan et al., 2009). Nevertheless, nursing interns consider the internship period one of the most stressful periods in nursing education (Walker and Costa, 2017). Previous research has hypothesized that the main stressors among Chinese nursing interns may be a lack of knowledge and skills as well as a fear of medical errors due to inadequate preparation (Li et al., 2020), which exacerbates their anxiety and depressive symptoms. Liu et al. (2019) found that Chinese undergraduate nursing interns were required to make career choices at the end of their internship, such as joining the nursing team to become clinical nurses, continuing their studies, or even leaving the nursing profession (Liu et al., 2019). The nationwide nursing qualification examination (pass rate of approximately 40%) during the process of job hunting also adds to the competitive pressure among Chinese nursing students (Yi et al., 2022). A previous study found that psychological distress among Chinese nursing interns was much higher than among experienced nurses (Chen and Hung, 2014).

However, to date, most studies have focused on undergraduate nursing interns; evidence on the prevalence of mental health problems and relative risk factors among higher vocational nursing interns is limited. To our knowledge, no studies have compared the mental health status of the two categories of higher vocational nursing students during their internships. In China, vocational nursing students spend 2 years in school, less than undergraduate students. Many studies have demonstrated that highly educated nurses can improve patient outcomes (Cho et al., 2018; Harrison et al., 2019); therefore, there is an increasing demand for highly educated nurses. Extensive studies conducted in China have revealed that nurses with a baccalaureate degree are the most needed in medical institutions (Liu et al., 2021). Meanwhile, national data showed that students recruited for advanced diploma and baccalaureate degree programs accounted for 39.36% and 10.09% of the total in 2017, respectively, which increased dramatically 10 years ago (Fu et al., 2022). Consequently, compared to undergraduate nursing interns, nursing interns from higher vocational colleges may experience greater pressure to find jobs, which might trigger anxiety and depressive symptoms in this vulnerable population. Additionally, 2-year college nursing students who graduated from secondary vocational schools had worse learning and coping patterns than 3-year college nursing students. Thus, internships may cause more emotional stress and place pressure on 2-year college nursing students who are younger student populations, potentially resulting in depression and anxiety problems (Chen et al., 2015).

This study aimed to explore the prevalence and risk factors of depressive and anxiety symptoms among higher vocational nursing interns in China and to provide data support for developing targeted interventions for this population to help them cope with mental health problems during their internships. It was predicted that (i) the prevalence of depressive and anxiety symptoms among higher vocational nursing interns will be high; (ii) two-year college nursing students will be more likely to have depressive and anxiety symptoms during internships than 3-year college nursing students; and (iii) depressive and anxiety symptoms will also be significantly associated with family support and hospital types among higher vocational nursing students during internships.

## 2 Materials and methods

### 2.1 Study design

This was a cross-sectional study. The study was performed in accordance with the guidelines of the Declaration of Helsinki and approved by the Ethics Committee of the the First Affiliated Hospital of Ningbo University.

### 2.2 Participants

From October 2023 to December 2023, nursing students on internships were consecutively recruited from a higher vocational college in Ningbo, Zhejiang Province. The target population of this study was nursing interns who had at least 5 months internship training period. A convenience sampling method was applied. The inclusion criteria were as follows: (1) aged 18 and above; and (2) never been diagnosed with depression, anxiety, and other mental illnesses that would interfere with completion of the measures, according to their latest mental health records conducted by the college. Signed informed consent was obtained from all participants. Eligible participants were administered standardized questionnaires, including all measures, and their sociodemographic data were provided. The sample size was determined based on the expected prevalence of depressive symptoms. Previous studies reported a prevalence of approximately 30%–40% (Chan et al., 2009; Chen et al., 2015). Using an expected rate of *P* = 35%, a two-sided significance level of α = 0.05, an allowable error of *d* = 0.05, and a design effect of 1.5, the minimum required sample size was calculated as 525. Ultimately, 501 eligible participants were included due to the constraints of the internship cycle. The observed prevalence of depressive symptoms was 36.7%, with a 95% confidence interval of ± 4.24%, which was close to the preset accuracy target.


$$\text{n}=\frac{{\left(1.96\right)}^{2}\times 0.35\times \left(1-0.35\right)}{{\left(0.05\right)}^{2}}\approx 525$$

### 2.3 Data collection

We used an online-based survey via the WeChat-based survey program “Questionnaire Star” to collect data. To ensure survey quality, investigators provided participants with clear guidance covering the purpose, methods, procedures, background, expected duration, and data involved. In addition, the system monitored completion time, and questionnaires submitted too quickly, suggesting careless responses, were flagged for later review.

### 2.4 Measurements

#### 2.4.1 Depressive symptoms

The Chinese version of the Self-Rating Depression Scale (SDS) was employed to assess depressive symptoms. The SDS is a 20-item self-report measure used to assess severity of depression. Participants rated each item in accordance with the frequency of a certain symptom within 7 days on a 4-point scale from one (none or little of the time) to four (most or all of the time). The final index score was converted by multiplying the raw score by 1.25 and then rounding off to decimal places. Depression severity was divided by index score into no depression (score < 50), mild depression (score 50–59), moderate depression (score 60–69), and major depression score ≥70) (Zung, 1965). The Chinese version of SDS has shown good validity and reliability (Tang et al., 2019). The Cronbach’s α was 0.83 in this study. The presence of depressive symptoms was defined as a total score of ≥52 points in the SDS in this study according to the Chinese norm.

#### 2.4.2 Anxiety symptoms

We employed the Chinese version of the Self-Rating Anxiety Scale (SAS) to assess anxiety symptoms. The SAS is a 20-item self-report measure used to assess the presence of anxiety symptoms. Participants rated each item in accordance with the frequency of a certain symptom within 7 days on a 4-point scale from one (none or a little of the time) to four (most or all of the time). The final index score was converted by multiplying the raw score by 1.25 and then rounding off to decimal places. Anxiety severity was divided by index score into no anxiety (score < 50), mild anxiety (score 50–59), moderate anxiety (score 60–69), and major anxiety (score ≥ 70) (Zung, 1971). It has been widely used in China, and studies have confirmed good validity and reliability of the Chinese version of the SAS (Jiang et al., 2021). The Cronbach’s α was 0.87 in this study. The presence of anxiety symptoms was defined as a total score of ≥50 points in the SAS in this study according to the Chinese norm.

#### 2.4.3 The growth background

Growth background was assessed using the UPI (University Personality Inventory) Supplementary Information scale (Supplementary Material). Family function and poverty were measured through participants’ subjective evaluations. Family function focused on the quality of interpersonal relationships within the nuclear family, including marital, parent–child, and sibling interactions. Responses indicating alienation, strain, or conflict were classified as low family function. Family intactness referred to the presence of both parents; divorce, recombined families, or the death of one or both parents were defined as not intact. In China, third-class hospitals—classified as the highest-level institutions with ≥500 inpatient beds, comprehensive specialty coverage, and integrated roles in clinical service, medical education, and scientific research—serve as regional referral hubs for complex and critical illnesses, comparable to tertiary referral hospitals internationally.

### 2.5 Statistical analyses

Continuous variables in a normal distribution were presented as mean ± standard deviation (SD). Continuous variables in a non-normal distribution were presented as median [lower quartile-upper quartile (IQR)]. Categorical variables were described as percentages (%). Prevalence rates of depressive, and anxiety symptoms with 95% confidence intervals (CI) are given as the proportion. The differences between 3-year college students and 2-year college students for continuous and categorical variables were estimated using Students’ *t*-test and the *X*^2^ test, respectively. Logistic regression models were used to examine the association between characteristics and depressive or anxiety symptoms with unadjusted and adjusted for covariates to test the stability of the results. Given that the objective of this study is exploratory—namely, to estimate the effects—variables with established or plausible clinical relevance will therefore be included in the model *a priori*, and the model retains variables irrespective of their statistical significance. Standardized coefficients were calculated to identify the most influential variable. Statistical analyses were performed using SPSS (version 22.0; SPSS Inc., Chicago, IL, USA). *P*-values were considered statistically significant at *P* < 0.05.

## 3 Results

**FIGURE 1:**
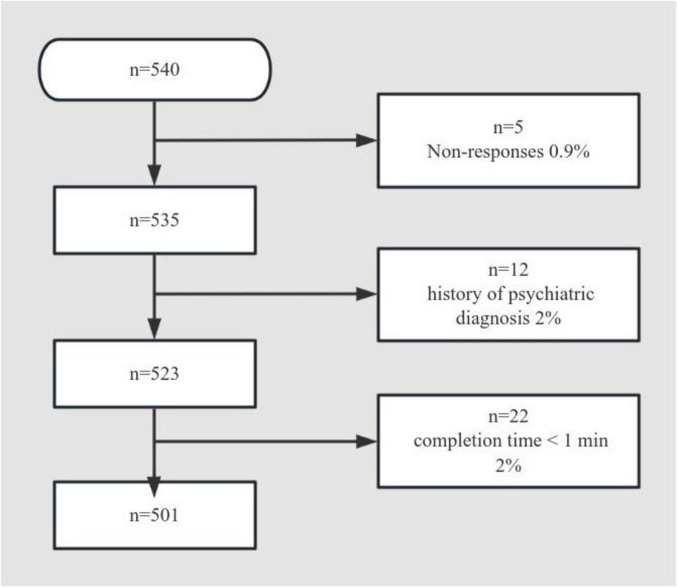
Participant flow diagram.

The study achieved a 92.8% response rate after excluding incomplete or invalid questionnaires, with 501 valid responses ultimately analyzed from the 540 vocational nursing students initially recruited (See Figure 1). Descriptive data of the sample are presented in Table 1. Most participants were 3-year college students, female, had intact families and high family function, and did not live in poverty. More than 60% of the sample came from rural areas and one-child families. Nearly half of the participants had internships in third-class hospitals. The overall prevalence of depressive and anxiety symptoms were 36.73% (95% CI: 32.72–41.24%) and 19.56% (95% CI: 16.26–23.31%), respectively. Among 3-year college students, the prevalence of depressive and anxiety symptoms were 33.52% (95% CI: 28.63–38.67%) and 11.45% (95% CI: 8.28–15.23%), respectively. Among 2-year college students, the prevalence of depressive and anxiety symptoms were 45.45% (95% CI: 37.14–54.03%) and 39.86% (95% CI: 8.28–15.23%), respectively. The prevalence of depressive symptoms (*P* = 0.012) and anxiety symptoms (*P* < 0.001) was significantly higher among 2-year college students than among 3-year college students.

**TABLE 1:** Sample characteristics of participants and univariate analysis of variables related to different program.

Variables	Total (*n* = 501)	Program		*P*-value
		Three-year college student (*n* = 358)	Two-year college student (*n* = 143)	
Mean ± SD				
Age, (years)	20.49 ± 0.71	20.60 ± 0.63	20.20 ± 0.810	<0.001
*N* (%)				
Sex				0.752
Male	38 (7.58)	28 (7.82)	10 (6.99)	
Female	463 (92.42)	330 (92.18)	133 (93.01)	
Residential type				0.554
Rural	308 (61.48)	223 (62.29)	85 (59.44)	
Urban	193 (38.52)	135 (37.71)	58 (40.56)	
Family is intact or not				0.995
No	77 (15.37)	55 (15.36)	22 (15.38)	
Yes	424 (84.63)	303 (84.64)	121 (84.62)	
Family function				0.001
High	470 (93.81)	344 (96.09)	126 (88.11)	
Low	31 (6.19)	14 (3.91)	17 (11.89)	
One-child family				0.021
No	323 (64.47)	242 (67.60)	81 (56.64)	
Yes	178 (35.53)	116 (32.40)	62 (43.36)	
Poverty or not			0.546	
No	491 (98.00)	350 (97.77)	141 (98.60)	
Yes	10 (2.00)	8 (2.23)	2 (1.40)	
Whether have a internship in third-class hospitals				<0.001
No	267 (53.29)	170 (47.49)	97 (67.83)	
Yes	234 (46.71)	188 (52.51)	46 (32.17)	
Depressive symptoms				0.012
No	317 (63.27)	238 (66.48)	79 (55.24)	
Yes	184 (36.73)	120 (33.52)	64 (45.45)	
Anxiety symptoms				<0.001
No	403 (80.44)	317 (88.55)	86 (60.14)	
Yes	98 (19.56)	41 (11.45)	57 (39.86)	

The associations between characteristics and depressive symptoms are shown in Table 2. After adjusting for covariates, younger age (odds ratio [OR]: 1.26), rural residence (OR: 1.80), low family function (OR: 2.96), internships in third-class hospitals (OR: 1.67), and enrollment as a 2-year college student (OR: 1.62) were associated with a higher prevalence of depressive symptoms. Standardized coefficient values further indicated that rural residence (1.33), low family function (3.73), and 2-year college status (1.08) were the strongest determinants of depressive symptoms.

**TABLE 2:** The relationship between sample characteristics and depressive symptoms.

Variables	Unadjusted			Adjusted		
	OR (95% CI)	β	*P-*value	OR (95% CI)	β	*P*-value
Age	1.16 (0.90–1.50)	0.190	0.264	1.26 (0.96–1.65)	0.295	0.103
Female	0.79 (0.40–1.55)	−0.944	0.492	0.87 (0.43–1.77)	0.550	0.704
Rural	1.91 (1.29–2.81)	1.459	0.001	1.70 (1.20–2.69)	1.326	0.004
Family is intact	0.91 (0.55–1.51)	−0.264	0.713	0.95 (0.56–1.62)	−0.144	0.847
Low family function	3.92 (1.80–8.52)	4.694	0.001	2.96 (1.32–6.62)	3.725	0.008
One-child family	0.87 (0.59–1.27)	−0.302	0.471	0.84 (0.56–1.26)	−0.380	0.395
Poverty	0.73 (0.19–2.85)	−1.719	0.648	0.59 (0.14–2.45)	−2.822	0.470
Have a internship in third-class hospitals	1.59 (1.40–1.85)	−1.074	0.004	1.67 (1.45–1.98)	−0.814	0.040
Two-year college	1.65 (1.11–2.46)	1.126	0.013	1.62 (1.05–2.52)	1.083	0.031

OR, odds ratio; CI, confidence interval.

The associations between the characteristics and anxiety symptoms are shown in Table 3. After adjusting for covariates, rural areas (OR: 3.34), low family function (OR: 2.91), an internship in a third-class hospital (OR: 1.42), and 2-year college student (OR: 4.23) were associated with an increased prevalence of depressive symptoms. Standardized coefficient values indicated that rural residence (2.73), low family function (3.67), and 2-year college status (3.23) were the strongest determinants of anxiety symptoms.

**TABLE 3:** The relationship between sample characteristics and anxiety symptoms.

Variables	Unadjusted			Adjusted		
	OR (95% CI)	β	*P-*value	OR (95% CI)	β	*P*-value
Age	0.65 (0.46–0.89)	−0.585	0.007	0.81 (0.56–1.160)	−0.278	0.244
Female	0.66 (0.31–1.40)	−1.687	0.278	0.75 (0.31–1.82)	−1.153	0.526
Rural	3.16 (1.84–5.41)	2.602	<0.001	3.34 (1.86–5.98)	2.726	<0.001
Family is intact	1.09 (0.60–2.00)	0.250	0.77	1.24 (0.63–2.44)	0.589	0.542
Low family function	5.05 (2.40–10.62)	5.564	<0.001	2.91 (1.23–6.85)	3.667	0.015
One-child family	1.26 (0.80–1.98)	0.490	0.326	1.11 (0.66–1.87)	0.218	0.705
Poverty	0.45 (0.06–3.61)	−4.303	0.453	0.32 (0.04–2.87)	−6.130	0.309
Have a internship in third-class hospitals	1.32 (1.19–1.52)	−2.292	<0.001	1.42 (1.25–1.72)	−1.734	0.002
Two-year college	5.13 (3.21–8.17)	3.664	<0.001	4.23 (2.49–7.20)	3.233	<0.001

OR: odds ratio; CI: confidence interval.

## 4 Discussion

This study revealed a high prevalence of depressive and anxiety symptoms among higher vocational nursing interns in China. Compared to 3-year college nursing students, 2-year college nursing students had a higher prevalence of depressive and anxiety symptoms during internships. Furthermore, among higher vocational nursing interns, depressive symptoms were significantly associated with younger age, rural area, low family function, having an internship in a third-class hospital, and being a 2-year college student, and anxiety symptoms were significantly associated with rural areas, low family function, an internship in a third-class hospital, and 2-year college student.

Our results are consistent with the findings of previous studies showing that psychological problems such as anxiety and depressive symptoms are common among Chinese undergraduate nursing interns (Li et al., 2020). Nevertheless, unlike the findings of the previous study that showed a higher prevalence of anxiety symptoms in nursing interns (Li et al., 2020), the results of our study revealed that depressive symptoms were the most common problem among nursing interns. A possible explanation for this finding is that the present study was conducted during the first half of the internship, whereas the previous one was conducted in the second half of the final year. This finding suggests that during the first half of the internship, more attention should be paid to depressive problems in higher vocational nursing interns.

We noted that 36.93% (95% CI: 32.72–41.24%) and 19.56% (95% CI: 16.26–23.31%) of nursing interns reported depressive and anxiety symptoms, respectively, which was much higher than the levels reported among the undergraduate nursing interns in China (Niu et al., 2023). Currently, most hospitals in China, especially third-class hospitals, are only willing to recruit undergraduate nursing students (Fu et al., 2022); We found that 36.93% (95% CI: 32.72–41.24%) of nursing interns reported depressive symptoms and 19.56% (95% CI: 16.26–23.31%) reported anxiety symptoms, proportions consistent with prior research. For instance, Zeng et al. (2019) reported a prevalence of depressive symptoms at 28.7% (95% CI: 24.9%–32.5%), slightly lower than the rate observed in this study. Compared with previous investigations that primarily examined undergraduate nursing students, the present study identified markedly higher prevalence rates of depressive and anxiety symptoms among 2- and 3-year vocational nursing students (Yi et al., 2022; Niu et al., 2023). This discrepancy may stem from the shorter duration of vocational programs—1–2 years less than undergraduate programs—which compresses theory, clinical practice, and assessment into a tighter timeframe, thereby increasing academic stress. Furthermore, in China, most hospitals—particularly third-class hospitals—prefer to employ nurses with bachelor’s degrees (Fu et al., 2022). As a result, vocational nursing interns experience greater employment pressure during clinical placements than their undergraduate counterparts. Thus, Chinese higher vocational nursing interns experience more pressure regarding job pursuit during the internship than undergraduate nursing interns. However, we could not directly compare the levels of depressive and anxiety symptoms between undergraduate nursing interns and higher vocational nursing interns. Future studies should be conducted to compare the prevalence of mental health problems among these two categories of nursing interns.

The results of this study are consistent with those of previous studies conducted in other developing countries. A prior study conducted in the Kingdom of Saudi Arabia found that 18.5% and 11.1% of participants had severe to extremely severe depression and extremely severe anxiety, respectively (Shdaifat et al., 2020). Another study conducted in Egypt revealed that more than 70% of participants reported severe anxiety (Rashwan et al., 2021). This result can be attributed to the inconsistent survey tools used in the investigation. Compared with nursing interns in developed countries, those in developing countries seem to be more likely to have mental problems (Zhang et al., 2021). One possible reason for this is that nurse density in developing countries is far below that in developed countries; thus, insufficient nurse staffing would directly increase the workload of nursing interns and have a negative influence on their mental health (Lu et al., 2012). Relevant departments in developing countries should pay special attention to the psychological distress among nursing interns. However, the prevalence of depressive symptoms in this study was significantly lower than the 52% reported in a previous meta-analysis conducted during the COVID-19 pandemic (Mulyadi et al., 2021). This finding suggests that depressive symptom prevalence among nursing students was higher in the early stages of the global pandemic.

The key finding of this study was that 2-year college nursing students were more likely to have depressive and anxiety symptoms during their internships than 3-year college nursing students. Similar results have also been observed in international nursing education systems, where comparisons between Chinese 2-year college nursing students and international counterparts can serve as a benchmark for distinguishing “accelerated” and “traditional” systems. International research further suggests that “shorter duration and faster pace” education models are generally associated with higher mental health risks, even though the structural design of programs differs. This phenomenon is evident not only in China but also in the United States, Brazil, and other countries where “accelerated nursing education” or “non-traditional” systems are implemented (Youssef and Goodrich, 1996; Furegato et al., 2010). There are several possible explanations for these results. First, 2-year college nursing students were younger and had only completed 9 years of general education; therefore, they were less mentally resilient than 3-year college nursing students. The experiences of caring for patients, interacting with patients and medical professionals, and dealing with death may cause greater depression and anxiety symptoms among younger nursing interns (Shikai et al., 2009). Second, compared with 3-year college nursing students, 2-year college nursing students spend fewer years in college taking theoretical courses; therefore, they are more likely to lack knowledge and skills, which may be one of the main stressors among Chinese nursing interns (Li et al., 2020). Prior studies conducted in China found that academic stress was strongly associated with depressive and anxiety symptoms among young nursing students (Xu et al., 2014; Dong et al., 2023). Time pressure, role conflict, and heavy learning burdens imposed by compressed education systems are key factors influencing psychological well-being. Consequently, the mental health risks faced by 2-year college nursing students are not unique to China but represent a common challenge of the global “compressed education system” model. Third, an increasing number of hospitals in China prefer to employ 3-year college nursing students instead of 2-year college nursing students (You et al., 2015), because they are more mature and have a stronger knowledge base. Thus, 3-year college nursing students need to face a more competitive work environment during the internship. Another previous study demonstrated that a competitive work environment was found to cause anxiety and depressive symptoms among Chinese vocational nursing interns (Chen et al., 2015).

The findings of this study align with those of Lei and Shen (2015), who discovered that urban areas offer better educational resources, enabling individuals to develop more comprehensive coping skills and resilience. These skills provide certain advantages in managing nurse-patient conflicts, thereby reducing the likelihood of psychological problems among nursing interns. Furthermore, the findings of this study revealed that nursing interns in third-class hospitals were more prone to experiencing symptoms of depression and anxiety compared to their counterparts in lower-level hospitals. The results were consistent with previous studies in China, which indicated that nurses in tertiary hospitals face greater mental health risks. This may be related to higher work intensity, increased risk of workplace violence, and significant challenges in occupational role adaptation encountered by nurses in tertiary hospitals (Yao et al., 2004). A previous study suggested that nursing interns in third-class hospitals are at a higher risk of encountering psychological workplace violence (WPV), which can be attributed to increased patient demands and higher treatment expectations. Higher vocational nursing interns may struggle to meet these demands due to their limited knowledge and skills (Yu et al., 2023). There is compelling evidence demonstrating the negative psychological consequences of WPV on nursing interns (Hopkins et al., 2018). In addition, nursing interns with low family functioning had higher levels of depressive and anxiety symptoms, similar to other research results (Quinn and Peters, 2017). During their internships, family support can help nursing students make a smooth transition out of college, which may relieve depressive and anxiety symptoms.

This study had several strengths. First, this is the first study, to the best of our knowledge, to explore the mental health problems and related factors among nursing interns in China. Second, participants’ mental health status was measured using validated measurement tools with good reliability. Second, the multivariate analysis may have caused type-I error inflation.

However, this study had several limitations that need to be considered. First, the timing of the survey was in the middle of the internship; therefore, interns had minimal clinical experience. It may have been preferable to wait until the end of the internship to allow for greater clinical exposure. Second, because this was a cross-sectional study, our results do not show a causal relationship. Third, having all interns in one higher vocational college at the same time was convenient, and only a subset of the higher vocational nursing intern population from Ningbo was included. Therefore, the generalizability of our findings may be limited. Fourth, the results were based on the participants’ self-reported scores, which could have caused selection bias.

## 5 Conclusion

The findings of this study suggest that higher vocational nursing students in China are likely to develop depressive and anxiety symptoms during internships, and 2-year college nursing students had a higher prevalence of depressive and anxiety symptoms than 3-year college nursing students. Our findings provide guidance for promoting mental health among higher vocational nursing interns, especially those 2-year college students. Specific intervention recommendations include: (1) Comprehensive psychological health screening should be conducted for nursing students before the commencement of their internship to identify individuals at risk, thereby enabling early recognition and professional intervention. (2) A social support system should be established, such as a mentoring program jointly developed by hospitals and schools. Regular symposia should also be organized to allow students to express concerns, while targeted guidance in professional skills—including communication and effective feedback—should be provided (Fang et al., 2020). (3) A psychological adjustment training program designed to improve workplace adaptability should be implemented on the eve of the internship, with mindfulness training serving as a key intervention strategy (Aloufi et al., 2021).

## Data Availability

The raw data supporting the conclusions of this article will be made available by the authors, without undue reservation.
