# Correction: Depressive and anxiety symptoms among higher vocational nursing interns in Ningbo, China: a cross-sectional study

**DOI:** 10.3389/fpsyg.2026.1922362

**Published:** 2026-07-22

**Authors:** Yuchen Ying, Xiaomei Chen, Li Wang

**Affiliations:** 1 Ningbo College of Health Sciences, Ningbo, Zhejiang, China; 2 Department of Psychosomatic Medicine, The First Affiliated Hospital of Ningbo University, Ningbo, Zhejiang, China

**Keywords:** higher vocational nursing students, nursing intern, depressive symptoms, anxiety symptoms, cross-sectional study

The order of authors in the author list of the published paper was erroneously given as:

Li Wang^1†^, Xiaomei Chen^2†^ and Yuchen Ying^1,2*^

The correct author list reads:

Yuchen Ying^1,2†^, Xiaomei Chen^2†^ and Li Wang^1*^

Author Yuchen Ying was erroneously assigned as corresponding author. The correct corresponding author is Li Wang.

Author Yuchen Ying was erroneously omitted as equal contributing author.

The funder General Research Project (Higher Education) of Zhejiang Provincial Educational Science Planning-Research on Post Competency Evaluation of Graduates majoring in Geriatric Health Care and Management Under the Background of Active Aging, [2023SCG135] to Yuchen Ying was erroneously omitted. The correct **Funding** statement is as follows:

The author(s) declared that financial support was received for this work and/or its publication. This article received funding from General Research Project (Higher Education) of Zhejiang Provincial Educational Science Planning-Research on Post Competency Evaluation of Graduates majoring in Geriatric Health Care and Management Under the Background of Active Aging, [2023SCG135] to Yuchen Ying.

The original version of this article has been updated.

