# Supplementary material for: Depressive and anxiety symptoms among higher vocational nursing interns in Ningbo, China: a cross-sectional study
Source: Front Psychol. 2026 Jan 22;16:1450071. doi: 10.3389/fpsyg.2025.1450071 (PMC12872921; doi:10.3389/fpsyg.2025.1450071)
Supplement: Supplementary file 1 [file Supplementary_file_1.docx]

**Appendix**

University Personality Inventory Supplementary Information Scale

1. Gender (Sex):

A. Male

B. Female

1. Place of Origin:

A. Provincial Capital

B. Non-provincial Capital City

C. Town

D. Rural Area

1. Birth Order:

A. Only Child

B. Eldest

C. Middle

D. Youngest

1. Family Structure:

A. Intact

B. Single-parent

C. Blended Family

D. Orphan

1. Family Economic Status:

A. Affluent

B. Good

C. Average

D. Impoverished

1. Family Function:

A. Close-knit

B. Harmonious

C. Alienated

D. Tense

E. Conflict-ridden

1. History of Mental Illness:

A. None

B. Diagnosed in Primary School

C. Diagnosed in Junior High School

D. Diagnosed in Senior High School
